# Transcriptional changes in the aphid species *Myzus cerasi* under different host and environmental conditions

**DOI:** 10.1111/imb.12631

**Published:** 2020-01-13

**Authors:** P. Thorpe, C. M. Escudero‐Martinez, S. Eves‐van den Akker, J. I. B. Bos

**Affiliations:** ^1^ Cell and Molecular Sciences The James Hutton Institute Dundee UK; ^2^ Division of Plant Sciences School of Life Sciences, University of Dundee Dundee UK; ^3^ Department of Plant Sciences University of Cambridge Cambridge UK

**Keywords:** aphid host adaptation, laboratory environment, RNAseq, detoxification

## Abstract

Aphids feature complex life cycles, which in the case of many agriculturally important species involve primary and secondary host plant species. Whilst host alternation between primary and secondary host can occur in the field depending on host availability and the environment, aphid populations maintained as laboratory stocks generally are kept under conditions that allow asexual reproduction by parthenogenesis on secondary hosts. We used *Myzus cerasi* (black cherry aphid) to assess aphid transcriptional differences between populations collected from primary hosts in the field and those adapted to secondary hosts under controlled environment conditions. Transfer of *M*. *cerasi* collected from local cherry trees to reported secondary host species resulted in low survival rates. Moreover, aphids were unable to survive on the secondary host land cress, unless first adapted to another secondary host, cleavers. Transcriptome analyses of the different aphid populations (field collected and adapted) revealed extensive transcriptional plasticity to a change in environment, with predominantly genes involved in redox reactions differentially regulated. Most of the differentially expressed genes were duplicated and we found evidence for differential exon usage. Our data suggest that aphid adaptation to different environments may pose a major hurdle and leads to extensive gene expression changes.

## Graphical abstract

- Limited survival when aphids collected from primary hosts in the field are transferred to secondary hosts in a lab environment.
- Extensive transcriptional responses in aphids associated with a change in host and/or environment.
- Differential regulation of sets of genes, many of which are involved in detoxification/redox.

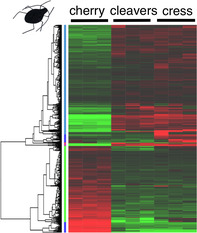

## Introduction

Aphids are phloem‐feeding insects that belong to the order Hemiptera. Insects within this order feature distinctive mouthparts, or stylets, that are used to pierce plant tissues and obtain nutrients from the plant phloem. One striking feature of the complex life cycle of about 10% of aphid species is the seasonal host switching between unrelated primary (winter) and secondary (summer) host plants, also called host alternation or heteroecy (Mordvilko, 1928; Williams and Dixon, 2007). Host alternating aphids predominantly use woody plants as their primary hosts, on which (overwintering) eggs are laid, from which the first generation of aphids, or fundatrices, emerge in spring. The fundatrices, and their offspring, reproduce by parthenogenesis (asexual reproduction), giving birth to live nymphs. Winged forms (alate) will migrate to secondary host plants over the summer months where the aphid populations will go through multiple parthenogenic generations. In autumn, sexual female and male aphids will reproduce sexually and overwintering eggs are laid on the primary host. Exceptions to this general life cycle exist, with some aphids for example having multi‐year cycles (Kennedy and Stroyan, 1959).

Heteroecy in aphids has independently arisen in different aphid lineages throughout evolutionary history (Moran, 1988) with monoecy (with the entire life cycle taking place on one plant species) on trees thought to be the ancestral state. Many different hypotheses explain the maintenance of heteroecy and driving factors described include nutritional optimization, oviposition sites, natural enemies, temperature tolerance, and fundatrix specialization (Moran, 1988). It is likely that switching between host plant species requires aphids to adapt to differences in host nutritional status as well as potential differences in plant defence mechanisms against insects. Host plant specialization in the pea aphid species complex is associated with differences in genomic regions encompassing predicted salivary genes as well as olfactory receptors (Jaquiéry *et al*., 2012). Moreover, adaptation of *Myzus persicae* to different secondary host plant species involves gene expression changes, including of genes predicted to encode for cuticular proteins, cathepsin B protease, Uridine 5′‐diphosphate (UDP)‐glycosyltransferases and P450 monooxygenases (Mathers *et al*., 2017). Aphid secondary hosts include many important agricultural crops and are generally more suitable for maintaining clonal (asexual) aphid laboratory stocks used for research experiments. To what extent aphid gene expression is affected upon collecting aphids from the field and adapting them to select secondary host plants in a laboratory environment remains unclear.

*Myzus cerasi*, or black cherry aphid, uses mainly *Prunus cerasus* (Morello cherry) and *Prunus avium* (sweet cherry), but also other *Prunus* species as primary hosts and several herbaceous plants (*Galium* spp., *Veronica* spp. and cruciferous species) as secondary hosts (Blackman and Eastop, 2000; Barbagallo *et al*., 2017). Infestation can cause significant damage on cherry trees, due to leaf curling, shoot deformation and pseudogall formation, and lead to fruit damage. Recently, we generated a draft genome for *M*. *cerasi*, providing novel insights into potential parasitism genes as well as genome evolution. The increasing availability of genomics resources for aphids, including *M*. *cerasi*, facilitates further understanding of aphid biology and plant infestation strategies.

To investigate *M*. *cerasi* transcriptional responses associated with adaptation to secondary host plants under a laboratory controlled environment, we made several attempts to establish populations on *Barbarea verna* (land cress) and *Galium aparine* (cleavers) using aphids collected from local primary hosts (cherry trees). We found that aphids collected from their primary host in the field differed in their ability to adapt to the secondary host plant species in a controlled environment, with no aphids surviving transfer to *B*. *verna* unless first adapted to *G*. *aparine*. Based on this we hypothesized that the transfer to a new host plant and/or environment (termed here the ‘host environment’) poses a major hurdle and this is most likely reflected by significant changes in gene expression. To test this, we compared the transcriptomes of *M*. *cerasi* aphids adapted under laboratory conditions to secondary hosts *G*. *aparine* and *B*. *verna* and only observed limited transcriptional changes. However, when comparing the transcriptomes of these adapted aphids to field collected aphids from primary hosts, we noted extensive transcriptional changes, especially with regards to predicted detoxification genes. The majority of differentially expressed genes were duplicated, implicating multigene families in aphid adaptation to host plants and/or environments.

## Results and Discussion

### Myzus cerasi *host adaptation under controlled laboratory conditions is associated with low survival rates*

**Figure 1:**
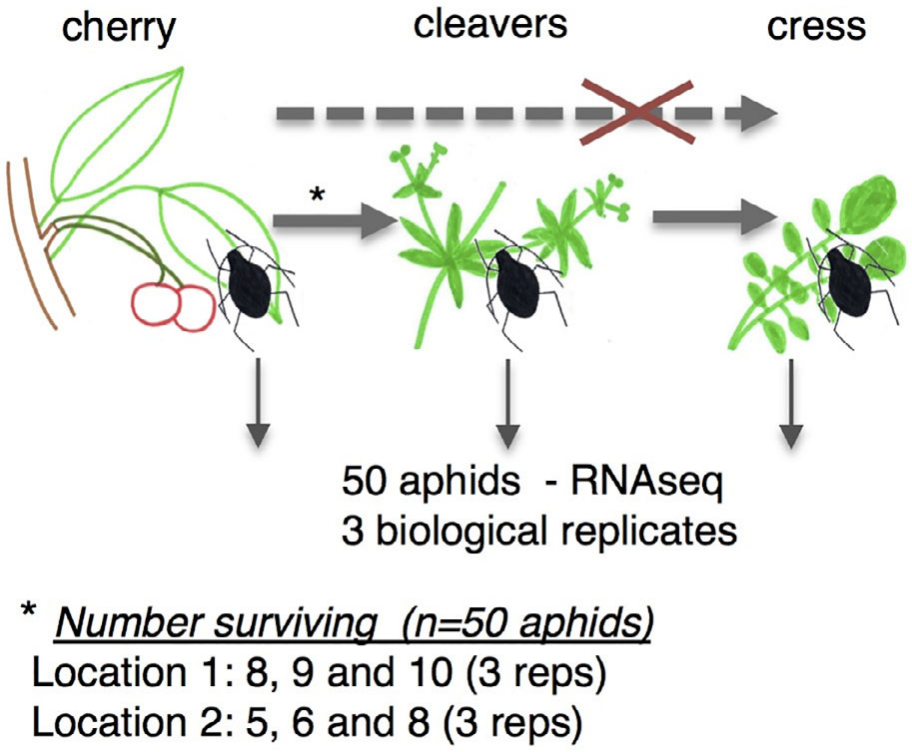
Schematic overview of host environment adaptation experiments and aphid survival rates. *Myzus cerasi* aphids were collected from cherry trees at two separate field locations. None of the aphids collected from cherry in the field was able to survive directly on *Barbarae verna* (land cress) plants. However, a 10–20% survival rate was recorded when aphids were moved onto *Galium aparine* (cleavers) in a controlled environment. The host adaptation experiments were performed in three biological replicates. [Colour figure can be viewed at wileyonlinelibrary.com]

When attempting to establish a colony of *M*. *cerasi* from populations occurring on local cherry trees, we observed differences in survival rates upon transfer to reported secondary host plant species under controlled plant growth conditions. Whereas aphids were unable to survive transfer from primary host cherry to land cress (*B*. *verna*), we observed a 10–20% survival rate upon transfer to cleavers (*G*. *aparine*) (Fig. 1).

However, once aphid populations were established on cleavers, individuals from this population were able to infest cress plants when exposed to a mixture of detached cleavers and cress leaves in cups. We performed similar field to lab host transfer experiments with aphids collected from cherry trees at two different locations in three independent replicates with similar results (Fig. 1). It should be noted that the *M*. *cerasi* populations on cherry predominantly consisted of apterous aphids of mixed‐age, which were used for transfer experiments, whereas in the field the alate aphids would migrate to new plants, including secondary host species. Whether apterous and alate *M*. *cerasi* aphids differ in their ability to adapt to new host plants and environments remains to be investigated, but it is possible that the low survival rates upon transfer reflects low adaptability of the apterous aphids. Moreover, the population on cherry is most likely a mix of genotypes that vary in their ability to colonize specific host species. Despite this, our data point to variation in the ability of *M*. *cerasi* collected from primary hosts in the field to infest two reported secondary hosts, and suggest that this aphid may be able to expand its host range upon colonizing certain host species (in this case cleavers). A similar change in host specialization has been reported in *Aphis gossypii* (cotton‐melon aphid), where cotton‐specialized aphids were unable to colonize cucumber without being first established on the intermediate host zucchini under controlled environment conditions (Wu *et al*., 2013). To what extent these findings represent a natural field environment is yet to be explored.

### Myzus cerasi *shows extensive transcriptional plasticity to a change in host plant and/or environment*

We assessed the changes that take place at the transcriptional level in *M*. *cerasi* when adapting the field‐collected aphids from cherry to secondary hosts in a controlled plant growth environment. Specifically, we sequenced the transcriptomes of *M*. *cerasi* populations collected from cherry (field conditions), and of aphids established over a 3‐week period on cleavers or cress (controlled environment) using RNA sequencing (RNAseq).

**Figure 2:**
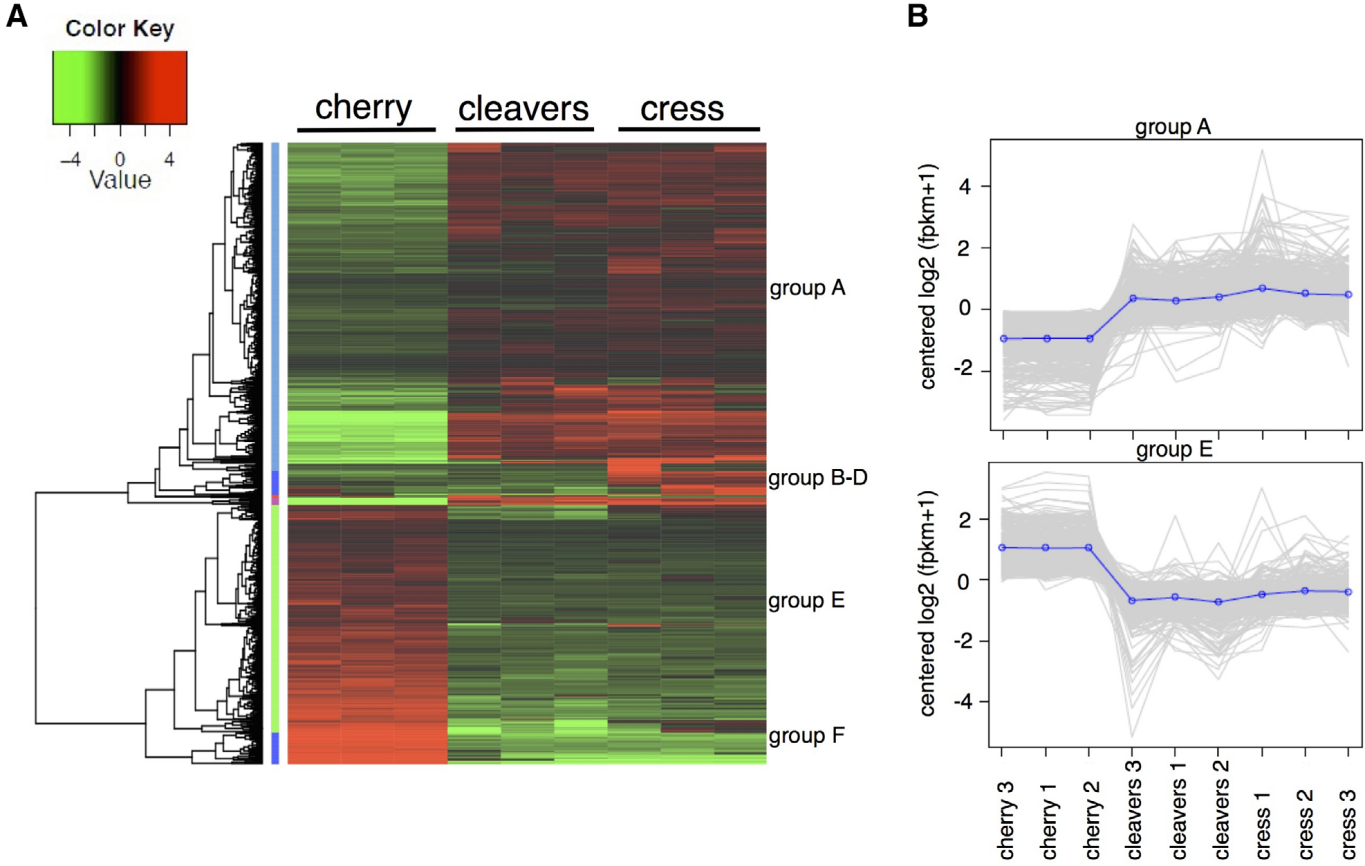
Clustering of differentially expressed genes across *Myzus cerasi* populations from primary (field) and secondary (controlled environment) hosts. (A) Cluster analyses of the 934 genes differentially expressed in *M*. *cerasi* populations from different host environments. (B) Expression profiles of the 493 coregulated genes in cluster A and of the 342 coregulated genes in cluster E. [Colour figure can be viewed at wileyonlinelibrary.com]

We performed differential gene expression analysis [log fold change >2, false discovery rate (FDR) *P* < 0.001] between the different aphid populations to identify gene sets associated with the different host plants and environments. Cluster analyses of the aphid transcriptional responses from this and previous work reporting on differential aphid gene expression in head vs. body tissues (Thorpe *et al*., 2016) revealed that the overall expression profiles could be distinguished based on the aphid tissue used for sample preparation as well as the host plant and environment (Fig. S1A). Whilst the tissue‐specific transcriptomes can be more clearly differentiated, principal component analyses showed a separation between the whole aphid transcriptomes associated with the primary host in field conditions and the different secondary hosts in controlled conditions (Fig. S1B). Overall, we identified 934 differentially expressed genes by comparing the different datasets for each of the aphid populations (Fig. 2A, Table S1). A heat map of these 934 genes shows that gene expression profiles from aphids adapted to secondary hosts (cleavers and cress) and maintained in a controlled environment are more similar to each other than to the gene expression profiles of aphids collected from primary hosts in the field (Fig. 2A). Co‐expression analyses reveals six main clusters of differentially expressed genes, two of which (A and E) contain the majority of genes (Fig. 2A, B). Cluster A contains 493 genes, which show higher expression in aphids maintained on secondary hosts in a controlled environment vs. those collected from primary hosts in the field, and cluster E contained 342 genes showing an opposite profile. Gene Ontology (GO) annotation revealed over‐representation of terms associated with oxido‐reductase activity in both clusters, as well as several terms associated with carotenoid/tetrapenoid biosynthesis in the case of cluster E (Table S2). The broader role of carotenoid synthesis genes in aphids, besides affecting body colour, remains largely unknown. However, in spider mites, carotenoids are essential for diapause induction (Bryon *et al*., 2017), and may potentially function as reactive oxygen species scavengers during oxidative stress (Zanga *et al*., 2018). The activation of different sets of oxidoreductases in clusters A and E could reflect that the aphid populations from primary hosts in the field vs. those from secondary hosts in a controlled environment need to cope with different plant responses and substrates associated with oxidative stress in the host. It should be noted that in our experimental set‐up with aphids collected both under field and controlled environment conditions, other factors in addition to the host plant species, or even host plant species × environment interactions, could account for the differences in gene expression across the aphid populations. For example, temperature regimes can affect aphid gene expression (Yang *et al*., 2015), and would fluctuate in a field environment but are constant in a controlled environment. Similarly light, humidity and other factors could impact aphid gene expression, but to what extent remains an open question.

**Figure 3:**
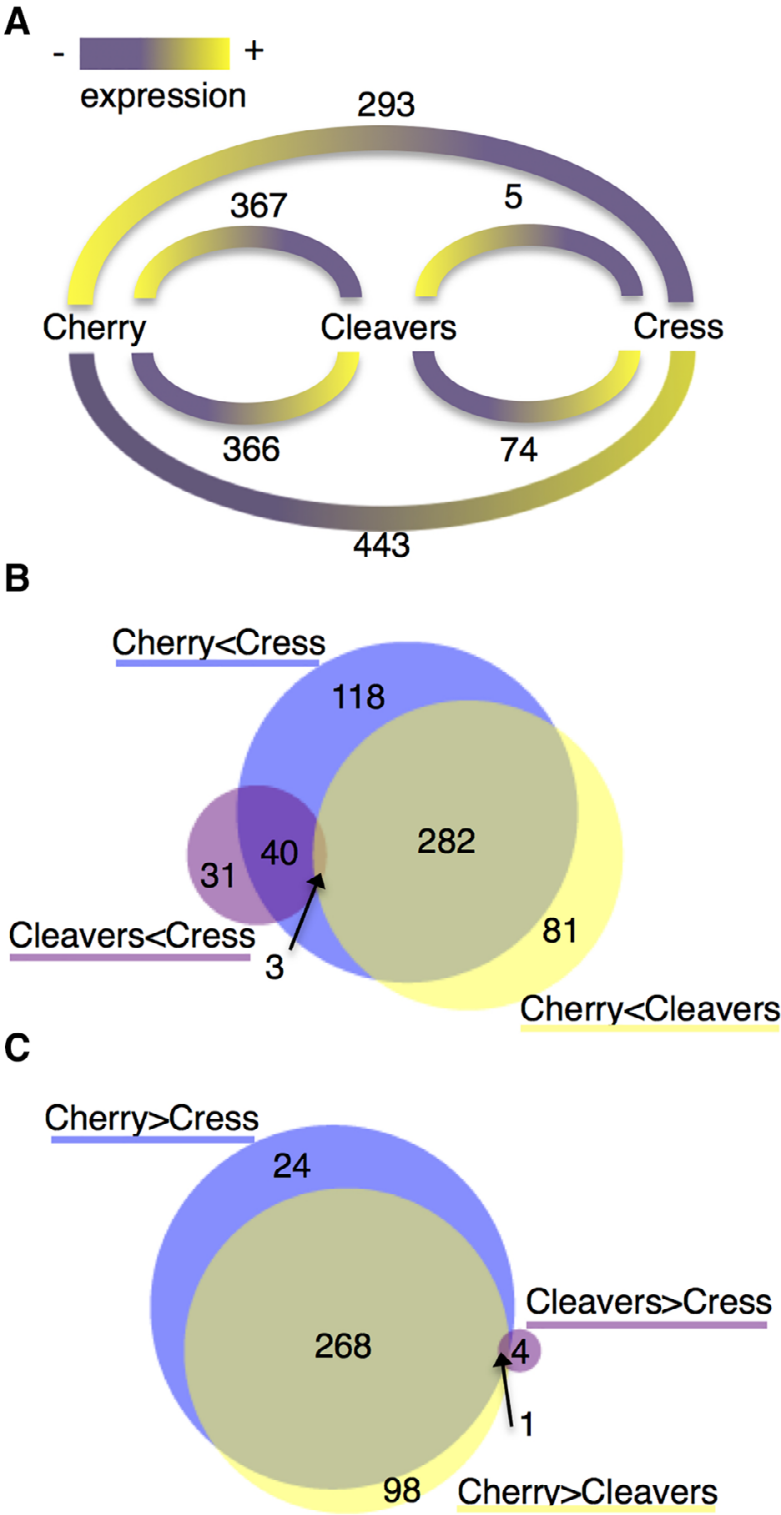
Differentially expressed genes in pairwise comparisons between the different *Myzus cerasi* populations. (A) Numbers of genes for each pairwise comparison between aphids collected from the different host species, cherry (field), cleavers (controlled environment) and cress (controlled environment). Yellow colour indicates a high level of expression, whereas purple colour indicates low expression in the different pairwise comparisons. (B) Venn diagram showing the overlap in differentially expressed gene sets that are expressed at lower levels in the aphids from the primary host cherry (field) compared to those collected from secondary hosts cleavers and cress (both in a controlled environment), and also expressed at lower levels in aphids from cleavers than those from cress. (C) Venn diagram showing the overlap in differentially expressed gene sets that are more highly expressed in the aphids from the primary host cherry (field) compared to those collected from secondary hosts cleavers and cress (both in a controlled environment), and also more highly expressed in aphids from cleavers than those from cress. [Colour figure can be viewed at wileyonlinelibrary.com]

To assess differential expression of *M*. *cerasi* genes across the different aphid populations we also analysed pairwise comparisons for differentially expressed gene sets. The largest set of differentially expressed genes (736) was found in comparisons between aphids from cherry (primary host, field) and cress (secondary host, controlled environment), with 443 genes more highly expressed in aphids from cress, and 293 more highly expressed in aphids from cherry (Fig. 3A). A total of 733 differentially expressed genes were found in comparisons of aphids from cherry (primary host, field) vs. cleavers (secondary host, controlled environment), with 367 genes more highly expressed in aphids collected from cherry and 366 genes more highly expressed in aphids collected from cleavers (Fig. 3A). The higher number of genes up‐regulated in the cress–cherry comparison may reflect the difficulties in adapting to this secondary host species, with *M*. *cerasi* unable to infest cress when collected from cherry (field).

A relatively small number of genes were differentially expressed between aphids collected from the two secondary hosts cleavers and cress (both grown under controlled conditions), with only five genes more highly expressed in aphids from cleavers, and 74 genes more highly expressed in aphids from cress (Fig. 3A). This suggests that *M*. *cerasi* shows limited transcriptional plasticity to a switch in secondary host environment, once adapted. Such limited transcriptional plasticity was also observed in our previous work where only a relatively small set of genes was differentially expressed in *M*. *persicae* and *Rhopalosiphum padi* when exposed to different host or non‐/poor‐host plants (Thorpe *et al*., 2018) as well in *M*. *persicae* when reared on different secondary host species (Mathers *et al*., 2017).

GO enrichment analyses of the 443 genes more highly expressed in aphids collected from cress (controlled environment) compared to those collected from cherry (field) show overrepresentation of genes predicted to be involved in various processes, including in heme binding (GO:0020037), tetrapyrrole binding (GO:0046906), monooxygenase activity (GO:0004497), oxidoreductase activity (GO:0016705), iron ion binding (GO:0005506) and hydrolase activity (GO:0016787) (Table 1). This set of 443 genes contains 282 of the 366 genes that are also more highly expressed in aphids from the other secondary host plant species, cleavers, with similar GO annotations (Fig. 3B; Table S4). The 293 genes more highly expressed in aphids collected from cherry (field) than those from cress (controlled environment) show over‐representation of genes predicted to be involved in oxidoreductase activity (GO:0016620, GO:0016903, GO:0055114, GO:001649) as well as other processes such as fatty‐acyl‐coenzyme A reductase (alcohol‐forming) activity (GO:0080019), interspecies interaction between organisms (GO:0044419), and symbiosis (GO:0044403) (Table S3). For the gene sets differentially expressed between aphids collected from cherry (field) and cleavers (controlled environment), GO enrichment analyses reveal that in reciprocal comparisons genes predicted to function in redox reactions are also over‐represented (Table S3).

Interestingly, amongst the 367 genes more highly expressed in aphids collected from cherry (field) compared to those collected from cleavers (controlled environment), we found that the majority of GO terms identified through enrichment analyses correspond to metabolic processes (Table S3). Of these 367 transcripts, 268 show similar expression differences in aphids collected from cherry (field) vs. those collected from cress (controlled environment), whereas 98 are specific to the comparison of aphids collected from cherry (field) vs. cleavers (controlled environment) (Fig. 3C). Whilst GO enrichment analyses showed over‐representation of genes involved in redox reactions in the set of 268 overlapping transcripts, the 98 transcripts specifically up‐regulated in aphids collected from cherry (field) vs. cleavers (controlled environment) show over‐representation in metabolic processes, and especially those associated with terpenoid/carotenoid biosynthesis, which are involved in aphid pigmentation (Table S5; Moran and Jarvik, 2010). Possibly this observation indicates that *M*. *cerasi* requires specific gene sets for pigmentation and feeding under specific host plant species and environmental conditions. Notably, we did not observe any noticeable change in aphid colour upon adapting aphids from primary hosts in the field to secondary hosts in the lab. Aphids featured a dark brown to black colour on all plant species tested (not shown), suggesting the differential regulation of carotenoid genes is not associated with aphid colour in this case but with other unknown physiological functions. The general over‐representation of differentially expressed genes involved in redox across the different *M*. *cerasi* populations most likely reflects different requirements for aphids under different host species and environmental conditions. Similar to our findings, comparative transcriptome analyses of pea aphids reared on different host plants also showed enrichment of genes with oxido‐reductase activity, potentially linked to detoxification (Eyres *et al*., 2016). In addition, gene annotation shows that several of our differentially expressed genes encode UDP‐glycosyltransferases and P450 monooxygenases, similar to several of the differentially expressed genes in *M*. *persicae* upon rearing aphids on different secondary hosts (Mathers *et al*., 2017). It is therefore possible that aphids employ at least some common strategies to adapt to new host environments.

To independently test whether select *M*. *cerasi* genes were differentially expressed in aphids collected from primary (field) and secondary (controlled environment) host plants, we repeated the collection of aphids from local cherry trees (separate site, location 2) and performed adaptation experiments to cleavers and cress. We selected 10 genes for independent validation of expression profiles by Quantitative Reverse Transcription PCR (RT‐qPCR). Five of these 10 genes were selected based on enhanced expression in aphids from cherry (field) compared to aphids from secondary (controlled environment) host plants, and another five genes for being more highly expressed in aphids from secondary (controlled environment) host plants compared to aphids from cherry (field). The genes selected based on higher expression in aphids from cherry (field) showed similarity to genes predicted to encode a peroxidase, RNA‐binding protein 14‐like, hybrid sensor histidine kinase response regulator, maltase isoform a and a lactase‐phlorizin hydrolase. The genes selected based on higher expression in aphids from secondary (controlled environment) hosts showed similarity to genes predicted to encode an unknown protein, a venom‐like protease, a thaumatin‐like protein, protein kintoun and a cytochrome P450. Except for the gene with similarity to a venom‐like protease, all genes showed a similar gene expression profile in both samples used for the RNAseq experiments and in the independently collected and adapted aphids from a different site, indicating that this gene set is consistently differentially expressed when *M*. *cerasi* was adapted from primary hosts in the field to secondary hosts in a controlled environment (Fig. S2). Most of these genes have predicted functions in detoxification, in line with our hypothesis that aphids require different sets of genes to deal with potential defensive plant compounds associated with different host plant and environmental conditions. To what extent our observations are associated with primary vs. secondary host factors or field vs. controlled environment factors is not clear. Notably, in *Hyalopterus persikonus* collected from primary and secondary host plant species in the field, a similar observation was made in that an extensive gene set associated with detoxification was differentially regulated (Cui *et al*., 2017).

### *Single nucleotide polymorphism (SNP) analyses suggest that an aphid subpopulation is able to adapt from primary hosts in the field to secondary hosts in a controlled environment*

We used the transcriptome dataset we generated here to compare the level of sequence polymorphisms between the aphid populations from the different primary (field) and secondary (controlled environment) host plants. Variants/SNPs were predicted by mapping the RNAseq dataset for each aphid population (cherry, cleavers and cress) to the *M*. *cerasi* reference genome for each condition, with only unique mapping being allowed. The number of SNPs within each 10 kb window was calculated (Table S6). The *M*. *cerasi* population from cherry has significantly more SNPs per 10 kb than the populations from both cleavers and cress when mapping reads back to the reference genome (*P* < 0.001, Kruskal–Wallis with Bonferroni post hoc correction). In contrast, the aphid populations from cleavers and cress showed no significant difference in the number of SNPs per 10 kb (*P* = 0.29, Kruskal–Wallis with Bonferroni post hoc correction). These results are consistent with the observation that the population of *M*. *cerasi* went through a bottleneck during the transfer from cherry in the field to cleavers in a controlled environment, but not when aphids were transferred from cleavers to cress (both in a controlled environment).

To gain an estimation of genetic diversity between the aphid samples obtained from each host, VCFtools (Danecek *et al.,* 2011) was used to return a π (the average number of nucleotide differences per site between the samples in all possible pairs, in the sample population) measured per 10 000 bases. The same window was used to assess SNPs per region. The overall π for aphids reared on cherry was smaller than that for the aphids reared on the secondary hosts (0.00009 vs. 0.0001 and 0.0001, respectively, Mann–Whitney *U*‐test), and the π of the cherry aphid population (field) was significantly different to the π of both the cress and cleavers populations (controlled environment), with *P* < 0.001 and *P* < 0.001, respectively. In contrast, the genetic diversity measurement for aphids reared on cress and cleavers (both in a controlled environment) was not significantly different (*P* = 0.21, Mann–Whitney *U*‐test). In addition, when comparing the ratio of heterozygous to homozygous SNPs from all aphid populations, we observed an increase in this ratio in aphids from the secondary hosts (controlled environment) when compared to those collected from cherry trees in the field (*P* = 0.001, *t*‐test). There was no difference in the ratio of heterozygous to homozygous SNPs in aphids from the two different secondary hosts (*P* = 0.85, *t*‐test), indicating that these populations are similar. Moreover, the ratio of observed homozygous sites vs. expected homozygous sites (*F*, inbreeding coefficient) ranged between 0.30 and 0.38 for aphids collected from cherry and between −0.13 and −0.46 for aphids adapted to secondary hosts under a controlled environment. These values are in line with relatively more heterogeneous populations on the secondary hosts vs. the primary host.

Based on these findings we propose that only a subpopulation of the primary host field population, which is more heterogeneous, may have been able to switch to secondary host plant species in a controlled environment. It should be noted that these data are based on RNAseq, and do not rule out the possibility of allele‐specific expression that may be associated with specific host/environment interactions. In addition, it is possible that abundant transcripts associated with a more dominant aphid population of less diverse aphids collected from cherry dominate the signal in our transcriptome dataset, which is based on Illumina sequencing (Illumina, San Diego, CA, USA). Further characterization of the *M*. *cerasi* (sub)populations using DNAseq will be required to gain further insight into adaptation of this aphid species to its hosts under different environmental conditions.

### *Limited differential expression of predicted *M*. *cerasi* effectors across field and adapted populations*

**Figure 4:**
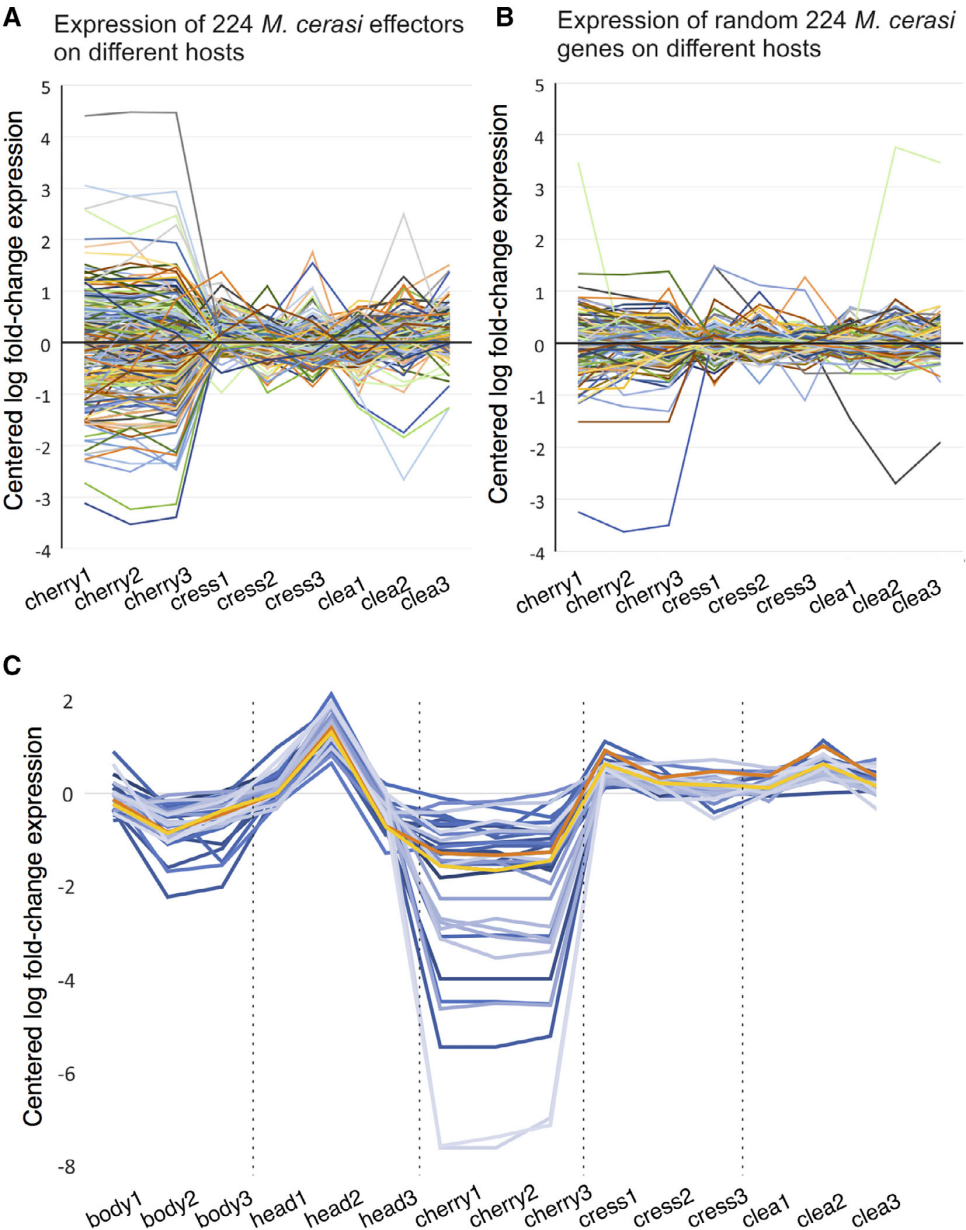
*Myzus cerasi* effector gene expression profiles across populations from different host environments. (A) Mean centred log fold‐change expression of 224 *M*. *cerasi* putative effectors across aphid populations from different host environments, including the primary host cherry, under field conditions, (cherry1–3), as well as cress (cress1–3) and cleavers (clea1–3), both in a controlled environment. (B) Mean centred log fold‐change expression of 224 randomly selected *M*. *cerasi* genes across aphid populations from different host environments, including cherry (cherry1–3), cress (cress1–3) and cleavers (clea1–3). (C) Identification of all other genes in the *M*. *cerasi* genome that are coregulated with the *Mc1:Me10‐like* pair based on a >90% Pearson's correlation across different populations (blue, *n* = 35). *Mc1* is indicated in orange and *Me10‐like* in yellow. [Colour figure can be viewed at wileyonlinelibrary.com]

Similar to plant pathogens, aphids deliver molecules, called effectors, inside their host to modify host cell processes and promote host susceptibility (Rodriguez and Bos, 2013). To investigate whether aphid effector proteins are potentially involved in *M*. *cerasi* host environment adaptation, we assessed whether predicted *M*. *cerasi* effector genes are differentially expressed in aphid populations collected from a primary host (field) or from secondary host plants (controlled environment). The 224 predicted *M*. *cerasi* effectors we previously identified show a wide range of expression levels across different interactions, with most expression variation in aphids collected from cherry under field conditions (Fig. 4A; Table S7). However, when assessing expression of a random non‐effector set of similar size, this expression variation in aphids was less pronounced (Fig. 4A). Despite the observed variation in expression patterns, we only found a small number of differentially expressed candidate effectors, mainly when comparing aphids collected from the primary (field) vs. secondary hosts (controlled environment). Specifically, 13 candidate effectors are more highly expressed in aphids from both secondary host species (controlled environment) compared to aphids from the primary host cherry (field), with one additional candidate effector more highly expressed in the case of aphids from cleavers compared to cherry only (Mca17157*|adenylate kinase 9‐like*; Table S6). Although these candidate effectors were mainly of unknown function, several show similarity to thaumatin‐like proteins and a venom protease. Interestingly, the candidate effector with similarity to the venom protease, Mca05785 [up‐regulated in secondary hosts (controlled environment)], is member of a venom protease gene family cluster that consists of four members (Mca05783, Mca05785, Mca05786, Mca05787, of which three are tandem duplications, and one is a proximal duplication). Three of these are predicted to encode secreted proteins, and all members show higher expression levels in aphids from secondary hosts under controlled conditions compared to aphids from primary host in the field, but this variation was below the log2 fold change cut‐off (Table S7). In addition, one candidate effector (similar to Mca07514*|RNA‐binding protein 14*) was differentially expressed when comparing aphids from the two secondary host plants (controlled environment), and five candidate effectors (Mca07285, Mca07514, Mca16980, Mca07516, Mca09259) were more highly expressed in aphids collected from cherry (field) compared to aphids from cleavers and/or cress (controlled environment) (Table S7).

### *Break‐down of aphid effector coregulation in the aphid population collected from primary hosts under field conditions*

In previous work we combined available transcriptome datasets for either *M*. *persicae* or *R*. *padi* to show that the expression of many aphid effector genes is tightly coregulated with the physically linked *Rp‐1‐like and Me10‐like* effector pair, under a number of conditions, pointing to a mechanism of shared transcriptional control (Thorpe *et al*., 2018). To assess this phenomenon in *M*. *cerasi* sequenced in previous work (under different conditions) and here, we performed a similar analysis by correlating the expression of the *Mc1* and *Me10‐like* pair to all other genes in the genome. A total of only 35 genes showed a Pearson's correlation of >90% across 15 available RNAseq libraries including the nine libraries from different host environments and six libraries from different aphid tissues (Fig. 4C). This number is much smaller compared to the set of coregulated genes in *R*. *padi* (213) and *M*. *persicae* (114) at the same threshold (Thorpe *et al*., 2018). Interrogating this further, it seems that the primary host cherry in the field is the reason so few coregulated genes were identified – genes that are tightly coregulated at every other stage appear to be considerably more variable in aphids collected from the primary host cherry in the field (Fig. 4C). Possibly, the diversity of the *M*. *cerasi* population collected from cherry in the field underlies the observed apparent break‐down in coregulation.

### *Differential exon usage in *M*. *cerasi* populations*

**Figure 5:**
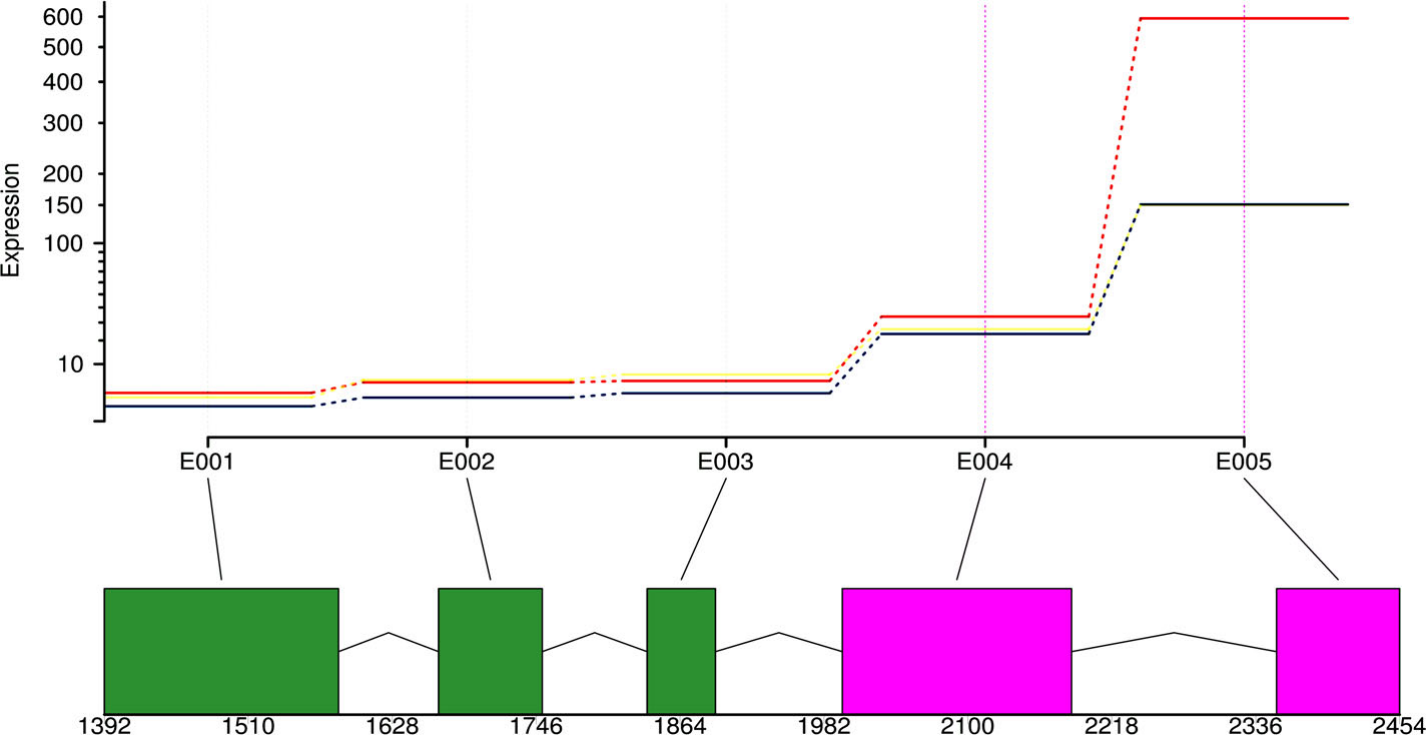
Graphical representation of differential exon usage observed in the gene Mca06436|*peroxidase‐like* in the transcriptome of *Myzus cerasi* populations from different host environments, including the primary host cherry, in the field (red line), as well as cress (yellow line) and cleavers (blue line), both in a controlled environment. The five different exons are indicated by E001–E005 and exons displaying significant differential expression are coloured pink. Numbers indicate nucleotide start and end positions of the different exons. The last exon shows four times greater expression in aphids collected from cherry in the field compared to those from cleavers or cress, both in a controlled environment. [Colour figure can be viewed at wileyonlinelibrary.com]

We also found evidence for differential exon usage when comparing the different aphid transcript datasets. Overall, 263 genes show significant differential exon usage when comparing aphid datasets associated with the different primary (field) and secondary hosts (controlled environment) (Table S8). These 263 genes contain 2551 exons, of which 443 show differential expression between aphid populations from primary hosts in the field vs. populations from secondary hosts in a controlled environment. No significant GO annotation is associated with these 263 genes. One example of differential exon usage in *M*. *cerasi* is peroxidase gene *Mca06436*, which contains five exons, two of which are significantly more highly expressed in aphids collected from primary hosts in the field (Fig. 5). This suggests that alternative splicing may be associated with the adaptation of the field population to the secondary hosts in a controlled environment.

### *The majority of *M*. *cerasi* genes differentially expressed across different host plant and/or environmental conditions are duplicated*

Interestingly, the majority of genes differentially expression across the three *M*. *cerasi* populations are duplicated (not single copy). For the genes up‐regulated in *M*. *cerasi* from cress (controlled environment) vs. cherry (field) only 14% are single copy, which is significantly lower than the percentage of single copy genes in a randomly selected set of genes (*P* < 0.001, Mann–Whitney *U*‐test). Moreover, for all sets of differentially expressed genes, the differentially expressed genes were more likely to be duplicated when compared to a background random gene set (*P* < 0.001; Table S9). To assess what types of gene duplication were represented within the differential expressed gene sets, 100 iterations of 100 randomly selected genes were conducted to obtain a background population. This yielded a mean and standard deviation for each duplication type from the parent gene population (normally distributed). A probability calculator (genstat) (VSN International: Hemel Hempstead, U.K.) was used to determine how likely the observed counts were to occur at random. Comparing the duplication events within our differentially expressed gene set to the random set revealed that most of the duplicated differentially expressed genes were within the ‘dispersed duplication’ category (*P* < 0.001) and that there was no significant difference in the occurrence of tandem or proximal gene duplications (*P* > 0.05) (Table S9). In contrast to predicted *M*. *cerasi* effectors, the differentially expressed genes identified in this study were not significantly further away from their neighbour in the 3′‐direction (*P* = 0.163, Mann–Whitney *U* Wilcoxon rank‐sum test), or their 5′ neighbour gene (*P* = 0.140, Mann–Whitney *U* Wilcoxon rank‐sum test) when compared to an equal sized random population (Fig. S3). Altogether our data suggest that *M*. *cerasi* multigene families may play an important role in adaptation to host plants and/or environmental conditions. This is in line with Mathers *et al*. (2017), who showed that duplicated genes play a role in adaptation of *M*. *persicae* to different secondary host species.

## Conclusion

Aphids feature complex life cycles, which in some cases involve alternation between summer and winter host plant species and need to cope with changes in the environment. Therefore, aphid populations in the field probably face different challenges than those maintained under controlled conditions and generally used for laboratory experiments. Here, we attempted to adapt *M*. *cerasi* from primary hosts in the field to secondary hosts in a controlled environment and studied transcriptional responses upon adaptation. We observed low survival rates of aphids in our adaptation experiments, suggesting that the switch from a primary host in the field to a secondary host in a controlled environment may pose a hurdle and potentially only specific subpopulations are able to adapt under the conditions tested. Comprehensive gene expression analyses of the aphid populations collected from primary hosts in the field and adapted to secondary hosts in a controlled environment revealed sets of detoxification genes that are differentially regulated and differential exon usage associated with a change in host plant and/or environmental conditions. Many of the differentially expressed genes are members of multigene families. In contrast, we find only limited transcriptional plasticity to secondary host switching under controlled conditions. Further research will be needed to dissect out which transcriptional responses are associated with either the switch from primary to secondary hosts and/or the transfer from the field to a controlled environment.

## Experimental procedures

### *Aphid collection and adaptation*

*M*. *cerasi* was collected in July 2013 from two separate locations in Dundee, UK. Populations on the selected branches consisted of mixed‐age, and predominantly apterous, aphids. Mixed‐age apterous aphids from cherry were flash frozen in liquid nitrogen upon collection (three replicates of 50 aphids per location). For adaptation to secondary host plants, 50 apterous aphids of mixed age were transferred to *G*. *aparine* (cleavers) or *B*. *verna* (land cress) detached branches placed in three replicate cup cultures per location. Aphid survival was assessed after 1 week. Then, five aphids of the surviving population on cleavers were moved to a fresh cup culture containing detached cleavers branches. Fresh plant material was added to the cups after 2 weeks. One week later 50 mixed‐age aphids per cup were flash frozen (aphids adapted to cleavers for RNAseq) and fresh cleavers branches together with land cress branches were added to the cups. One week after adding the land cress plant material, all cleavers material was removed and fresh cress branches were added and fresh plant material was regularly provided. Three weeks later 50 mixed‐age apterous aphids were collected per cup culture and flash frozen (aphids adapted to cress for RNAseq). Aphids were maintained in cup cultures in controlled environment cabinets at 18°C with a 16 h light and 8 h dark period.

### *RNA sample preparation and sequencing*

Aphid samples were ground to a fine powder and total RNA was extracted using a plant RNA extraction kit (Sigma‐Aldrich, St Louis, MO, USA), following the manufacturer's instructions. We prepared three biological replicates for *M*. *cerasi* collected from each host. RNA quality was assessed using a Bioanalyzer (Agilent Technologies, Santa Clara, CA, USA) and a Nanodrop (Thermo Scientific, Waltham, MA, USA). RNA sequencing libraries were constructed with an insert size of 250 bp according to the TruSeq RNA protocol (Illumina, San Diego, CA, USA), and sequenced at the previous Genome Sequencing Unit at the University of Dundee using Illumina‐HiSeq 100 bp paired end sequencing. All raw data are available under accession number PRJEB24338.

### *Quality control, RNAseq assembly and differential expression*

The raw reads were assessed for quality before and after trimming using fastqc (Andrews, 2010). Raw reads were quality trimmed using trimmomatic (Q22) (Bolger and Giorgi, 2015), then assembled using genome‐guided trinity (version r20140717) (Grabherr *et al*., 2011). transrate was run twice to filter out low supported transcripts (Smith‐Unna *et al*., 2015).

RNAseq assembly and annotation is available at DOI: 10.5281/zenodo.1254453. For differential gene expression, reads were mapped to the *M. cerasi* genome (Thorpe *et al*., 2018), per condition using star (Dobin *et al*., 2013). Gene counts were generated using bedtools (Quinlan and Hall, 2010). Differential gene expression analysis was performed using edger (Robinson *et al*., 2010), using log fold change >2, FDR *P* < 0.001 threshold. GO enrichment analysis was performed using blast2go (version 2.8, database September 2015; Conesa *et al*., 2005) using FDR 0.05. The genome annotations were formatted using genometools (Gremme *et al*., 2013) and subsequently htseq (Anders *et al*., 2015) was used to quantify exon usage. Differential exon expression was performed using dexseq FDR *P* < 0.001 (Anders *et al*., 2012). Heatmaps were drawn as described in Thorpe *et al*. (2018).

Gene duplication categories were used from Thorpe *et al*. (2018). Briefly, gene duplication analysis was performed using the mcscanx toolkit (Wang *et al*., 2012), which attempts to subdivide the types of gene duplication into the following categories by both (amino acid) sequence similarity and position in the genome: (1) single copy genes are defined as genes with no similarity to other genes within the thresholds used in mcscanx; (2) dispersed gene duplication is defined by genes that pass the thresholds for being classed as duplicated but are separated by greater than 10 nonrelated genes; (3) proximal gene duplication is defined by genes that pass the thresholds for being classed as duplicated but are separated by a maximum of 10 genes; (4) tandem duplications are duplication events that are next to each other; (5) segmental duplications are classed as either ‘whole genome duplication events’ or a subsection thereof. Those genes classed by mcscanx as dispersed, proximal, and tandemly duplicated will usually contain paralogous gene families, whereas those classed by mcscanx as segmental duplicated will usually contain segmental duplicated genes. In this paper we take into consideration both segmental and paralogous types of duplication.

A random population of genes was generated by running 100 iterations on a set of 100 randomly selected genes and their duplication types for subsequent statistical analyses. The script to generate random mean and standard deviation counts of genes assigned to a duplication class is available on Github (https://github.com/peterthorpe5/Myzus.cerasi_hosts.methods). Statistical analysis was performed using Probability Calculator in genstat (17th edition). The obtained value from the gene set of interest (differentially expressed genes across aphid populations) was compared to the distribution of the random test set. Datasets identified as being significantly different from the random population of genes did not significantly deviate from a normal distribution, thus the data were normally distributed. To assess the distances from one gene to the next, we generated an equal sized population of random genes (1020 and assessed their values for distance to their neighbouring gene in a 3′‐ and 5′‐ direction . The real and the random values were not normally distributed and were analysed in genstat (17th edition) using a nonparametric Mann–Whitney *U* Wilcoxon rank‐sum test.

For SNP identification, RNAseq data were mapped back to the reference genome using star (2.5.1b) with ‐outSAMmapqUnique 255, to allow only unique mapping (Dobin *et al*., 2013). SNPs were identified using freebayes (Garrison and Marth, 2012). vcftools ‐SNPdensity (0.1.15) (Danecek *et al*., 2011) was used on the resulting vcf files to identify SNPs per 10 kb. Extra vcf statistics were generated using rtg‐tools (https://github.com/RealTimeGenomics/rtg-tools). vcftools ‐window‐pi was used at the same 10 kb interval to obtain a measure of genetic diversity per aphid/host condition. The data were not normally distributed so a nonparametric test (Mann–Whitney) was used to statistically analyse the data. vcftools ‐het was used to investigate expected vs. observed heterozygosity.

To identify all other genes in the *M*. *cerasi* genome that are coregulated with the *Mc1:Me10‐like* pair, a Pearson's correlation was carried out using the data generated herein (nine libraries), and the RNAseq data previously generated of heads and bodies (Thorpe *et al*., 2018). A cut‐off of >90% was applied (Thorpe *et al*., 2018) to define tight coregulation.

### *Validation of expression profiles by qRT‐PCR*

Validation of the RNAseq experiment was completed with the Universal Probe Library (UPL) RT‐qPCR system (Roche Diagnostics, Basel, Switzerland). RNA samples analysed were those used for RNAseq analyses (aphid collections from location 1) as well as samples from aphids collected at a separate location (location 2) and adapted to secondary hosts (three biological replicates). For all experiments, aphid RNA was extracted using a RNeasy Plant Mini Kit (Qiagen, Hilden, Germany). RNA samples were DNAse treated with Ambion® TURBO DNA‐free™ (Ambion, Austin, TX, USA). SuperScript® III Reverse Transcriptase (Invitrogen, Carlsbad, CA, USA) and random primers were used to prepare cDNA. Primers and probes were designed using the predicted gene sequences generated in the RNAseq data analysis and the Assay Design Center from Roche, selecting ‘Other organism’ (https://lifescience.roche.com/en_gb/brands/universal-probe-library.html). Primers were computationally checked to assess if they would amplify one single product using emboss primersearch. Primers and probes were validated for efficiency (86–108%) before gene expression quantification; five dilutions of threefold for each primer pair‐probe were used for generating the standard curve. The 1:10 dilution of cDNA was selected as optimal for RT‐qPCR using the UPL system. Reactions were prepared using 25 μl of total volume, 12.5 μl of FastStart TaqMan Probe Master Mix (containing ROX reference dye) (Roche, Basel, Switzerland), 0.25 μl of gene‐specific primers (0.2 mM) and probes (0.1 mM). A Step‐One thermocycler (Applied Biosystems, Foster City, CA, USA) was set up as follows: 10 min of denaturation at 95°C, followed by 40 cycles of 15 s at 94°C and 60 s at 60°C. Relative expression was calculated with the delta‐delta cycle threshold (ΔΔCT) method with primer efficiency consideration. Three technical replicates were run per sample. Reference genes for normalization of the cycle threshold values were selected based on constant expression across different conditions in the RNAseq experiment. The reference genes were *Cell Division Cycle 42 (CDC42)‐Kinase* (Mca01274), *actin* (Mca10020) and *tubulin* (Mca04511). The fold change calculations were done by the ΔΔCT method and primer efficiency was taken into consideration.

## Supporting information


**Figure S1.** Transcriptome differences between *Myzus cerasi* populations from different host environments. Genome‐wide analysis of *M*. *cerasi* transcriptional responses to interaction with primary host cherry (field) or secondary hosts cress and cleavers (both in controlled environment), and comparison to previously published tissue‐specific transcriptome of dissected heads and bodies (Thorpe, Cock, and Bos, 2016). (A) Clustering of transcriptional responses reveals that *M*. *cerasi* gene expression is different in populations from the different host environments and also that expression in head and body tissues can be separated based on these analyses. (B). Principal component analysis. The top three most informative principal components describe approximately 75% of the variation, and separate the both the host species interaction data as well as tissue‐specific data well.
**Figure S2.** Validation of differential gene expression by Quantitative Rerverse Transcription PCR (RT‐qPCR). (A) Genes up‐regulated during cherry (field) vs. cleavers/cress (controlled environment) interactions in the *Myzus cerasi* population collected from location 1. (B) Genes up‐regulated during the cleavers/cress (controlled environment) vs. the cherry (field) interactions in the *M*. *cerasi* population collected from location 1. (C) Genes up‐regulated during cherry (field) vs. cleavers/cress (controlled environment) interactions in the *M*. *cerasi* population collected from location 2. (D) Genes up‐regulated during the cleavers/cress (controlled environment) vs. the cherry (field) interactions in the *M*. *cerasi* population collected from location 2. The validated genes up‐regulated during the cherry interactions were *peroxidase* (Mca14094‐Per), *protein kinase* (Mca07516‐PK), *RNA binding* (Mca07514‐RNAb), *maltase* (Mca25862‐Mal) and *lactase* (Mca19306‐Lac). Validated genes up‐regulated during the cress/cleavers interactions were *venom protein* (Mca05785‐Ven), *uncharacterized protein* (Mca06816‐UN), *unknown protein* (Mca06864‐UK), *cytochrome 450* (Mca22662‐c450) and *thaumatin* (Mca12232‐Thau). Blue and green series represent RT‐qPCR validation results and pale blue and pale green represent RNA‐sequencing (RNAseq) results. Error bars indicate standard error.
**Figure S3.** Heat maps graphically representing the log nucleotide distance from one gene to its neighbouring genes in a 3′‐ and 5′‐direction. Various gene categories are coloured and coded in the relevant keys. (A) and (B) Genetic distance heat map for predicted effectors, which were significantly further away from their neighbouring genes and thus in gene sparse regions (Thorpe *et al*., 2018). (C) and (D) Genic distances for differentially expressed genes identified in this study. These are not significantly further away from their neighbouring genes in either the 3′‐ or 5′‐direction.

**Table S1.** List of 934 differentially expressed *Myzus cerasi* genes across different host environments.
**Table S2** List of significant Gene Ontology terms associated with genes differentially expressed in clusters A and E (Fig. 2).
**Table S3.** List of significant Gene Ontology terms associated with genes differentially expressed across different *Myzus cerasi* host environments, corresponding to Fig. 3A.
**Table S4.** List of significant Gene Ontology terms associated with genes differentially expressed across different *Myzus cerasi* host environments, corresponding to Fig. 3B.
**Table S5.** List of significant Gene Ontology terms associated with genes differentially expressed across different *Myzus cerasi* host environments, corresponding to Fig. 3C.
**Table S6.** Overview of *Myzus cerasi* single nucleotide polymorphism data.
**Table S7.** List of 224 *Myzus cerasi* putative effectors and their expression levels across different host environments.
**Table S8.** List of *Myzus cerasi* genes showing differential exon usage across different host environments.
**Table S9.** Gene duplication types in *Myzus cerasi* genes differentially expressed across different host environments.

## Data Availability

All data are available under accession number PRJEB24338. The *M. cerasi* genome and annotation were downloaded from http://bipaa.genouest.org/is/aphidbase/ and DOI:10.5281/zenodo.1252934. All custom python scripts used to analyse the data use biopython (Danecek *et al*., 2011), and these scripts, as well as details on how they were applied for data analyses are available on https://github.com/peterthorpe5/Myzus.cerasi_hosts.methods, and DOI: 10.5281/zenodo.1254453.

## References

[imb12631-bib-0001] Anders, S. , Pyl, P.T. and Huber, W. (2015) HTSeq: a Python framework to work with high‐throughput sequencing data. Bioinformatics, 31, 166–169. 25260700 10.1093/bioinformatics/btu638 PMC4287950

[imb12631-bib-0002] Anders, S. , Reyes, A. and Huber, W. (2012) Detecting differential usage of exons from RNA‐seq data. Genome Research, 22, 2008–2017. 22722343 10.1101/gr.133744.111 PMC3460195

[imb12631-bib-0003] Andrews, S (2010) FastQC: a quality control tool for high throughput sequence data. Available at URL: http://www.bioinformatics.babraham.ac.uk/projects/fastqc.

[imb12631-bib-0004] Barbagallo, S. , Cocuzza, G.E.M. , Cravedi, P. and Shinkichi, K. (2017) In: *IP Case Studies: Decidious Fruit Tree Aphids*. *Aphids as Crop Pests*, Vol., 2nd edition. Wallingford: CABI International.

[imb12631-bib-0005] Blackman, R. and Eastop, V. (2000) In: Aphids on the World's Crops: An Identification and Information Guide. Wiley Chichester, U.K.

[imb12631-bib-0006] Bolger, A and Giorgi, F , Trimmomatic: A Flexible Read Trimming Tool for Illumina NGS Data, http://www.usadellab.org/cms/index.php [accessed January 2015].

[imb12631-bib-0007] Bryon, A. , Kurlovs, A.H. , Dermauw, W. , Greenhalgh, R. , Riga, M. , Grbić, M. *et al* (2017) Disruption of a horizontally transferred phytoene desaturase abolishes carotenoid accumulation and diapause in. Proceedings of the National Academy of Sciences of the United States of America, 114, E5871–E5880. 28674017 10.1073/pnas.1706865114 PMC5530703

[imb12631-bib-0009] Conesa, A. , Götz, S. , García‐Gómez, J.M. , Terol, J. , Talón, M. and Robles, M. (2005) Blast2GO: a universal tool for annotation, visualization and analysis in functional genomics research. Bioinformatics, 21, 3674–3676. 16081474 10.1093/bioinformatics/bti610

[imb12631-bib-0010] Cui, N. , Yang, P.C. , Guo, K. , Kang, L. and Cui, F. (2017) Large‐scale gene expression reveals different adaptations of Hyalopterus persikonus to winter and summer host plants. Insect Science, 24, 431–442. 28547891 10.1111/1744-7917.12336

[imb12631-bib-0011] Danecek, P. , Auton, A. , Abecasis, G. , Albers, C.A. , Banks, E. , DePristo, M.A. *et al* (2011) The variant call format and VCFtools. Bioinformatics, 27, 2156–2158. 21653522 10.1093/bioinformatics/btr330 PMC3137218

[imb12631-bib-0012] Dobin, A. , Davis, C.A. , Schlesinger, F. , Drenkow, J. , Zaleski, C. , Jha, S. *et al* (2013) STAR: ultrafast universal RNA‐seq aligner. Bioinformatics, 29, 15–21. 23104886 10.1093/bioinformatics/bts635 PMC3530905

[imb12631-bib-0013] Eyres, I. , Jaquiéry, J. , Sugio, A. , Duvaux, L. , Gharbi, K. , Zhou, J.J. *et al* (2016) Differential gene expression according to race and host plant in the pea aphid. Molecular Ecology, 25, 4197–4215. 27474484 10.1111/mec.13771

[imb12631-bib-0014] Garrison, E and Marth, G (2012) Haplotype‐based variant detection from short‐read sequencing. Available at: https://arxiv.org/abs/1207.3907 [accessed February 2016].

[imb12631-bib-0015] Grabherr, M.G. , Haas, B.J. , Yassour, M. , Levin, J.Z. , Thompson, D.A. , Amit, I. *et al* (2011) Full‐length transcriptome assembly from RNAseq data without a reference genome. Nature Biotechnology, 29, 644–652. 10.1038/nbt.1883 PMC3571712 21572440

[imb12631-bib-0016] Gremme, G. , Steinbiss, S. and Kurtz, S. (2013) GenomeTools: a comprehensive software library for efficient processing of structured genome annotations. IEEE/ACM Transactions on Computational Biology and Bioinformatics, 10, 645–656. 24091398 10.1109/TCBB.2013.68

[imb12631-bib-0017] Jaquiéry, J. , Stoeckel, S. , Nouhaud, P. , Mieuzet, L. , Mahéo, F. , Legeai, F. *et al* (2012) Genome scans reveal candidate regions involved in the adaptation to host plant in the pea aphid complex. Molecular Ecology, 21, 5251–5264. 23017212 10.1111/mec.12048

[imb12631-bib-0018] Kennedy, J.S. and Stroyan, H.L.G. (1959) Biology of aphids. Annual Review of Entomology, 4, 139–160.

[imb12631-bib-0019] Mathers, T.C. , Chen, Y. , Kaithakottil, G. , Legeai, F. , Mugford, S.T. , Baa‐Puyoulet, P. *et al* (2017) Rapid transcriptional plasticity of duplicated gene clusters enables a clonally reproducing aphid to colonise diverse plant species. Genome Biology, 18, 27. 28190401 10.1186/s13059-016-1145-3 PMC5304397

[imb12631-bib-0020] Moran, N.A. (1988) The evolution of host‐plant alternation in aphids: evidence for specialization as a dead end. American Naturalist, 132, 681–706.

[imb12631-bib-0021] Moran, N.A. and Jarvik, T. (2010) Lateral transfer of genes from fungi underlies carotenoid production in aphids. Science, 328, 624–627. 20431015 10.1126/science.1187113

[imb12631-bib-0022] Mordvilko, A.K. (1928) The evolution of cycles and the evolution of heteroecy (migration) in plant lice. Annals and Magazine of Natural History, 2, 570–582.

[imb12631-bib-0023] Quinlan, A.R. and Hall, I.M. (2010) BEDTools: a flexible suite of utilities for comparing genomic features. Bioinformatics, 26, 841–842. 20110278 10.1093/bioinformatics/btq033 PMC2832824

[imb12631-bib-0083] Rice, P. , Longden, I. and Bleasby, A. (2000) EMBOSS: The european molecular biology open software suite. Trends in Genetics, 16, 276–277. 10827456 10.1016/s0168-9525(00)02024-2

[imb12631-bib-0024] Robinson, M.D. , McCarthy, D.J. and Smyth, G.K. (2010) edgeR: a Bioconductor package for differential expression analysis of digital gene expression data. Bioinformatics, 26, 139–140. 19910308 10.1093/bioinformatics/btp616 PMC2796818

[imb12631-bib-0025] Rodriguez, P.A. and Bos, J.I.B. (2013) Toward understanding the role of aphid effectors in plant infestation. Molecular Plant‐Microbe Interactions, 26, 25–30. 23035915 10.1094/MPMI-05-12-0119-FI

[imb12631-bib-0026] Smith‐Unna, RD , Boursnell, C , Patro, R , Hibberd, JM and Kelly, S (2015) TransRate: reference free quality assessment of de‐novo transcriptome assemblies. Available at: https://www.biorxiv.org/content/10.1101/021626v1. 10.1101/gr.196469.115 PMC4971766 27252236

[imb12631-bib-0027] Thorpe, P. , Cock, P.J. and Bos, J. (2016) Comparative transcriptomics and proteomics of three different aphid species identifies core and diverse effector sets. BMC Genomics, 17(1), 172. 26935069 10.1186/s12864-016-2496-6 PMC4776380

[imb12631-bib-0028] Thorpe, P. , Escudero‐Martinez, C.M. , Cock, P.J.A. , Eves‐van den Akker, S. and Bos, J.I.B. (2018) Shared transcriptional control and disparate gain and loss of aphid parasitism genes. Genome Biology and Evolution, 10, 2716–2733. 30165560 10.1093/gbe/evy183 PMC6186164

[imb12631-bib-0029] Wang, Y. , Tang, H. , DeBarry, J.D. , Tan, X. , Li, J. , Wang, X. *et al* (2012) MCScanX: a toolkit for detection and evolutionary analysis of gene synteny and collinearity. Nucleic Acids Research, 40, e49. 22217600 10.1093/nar/gkr1293 PMC3326336

[imb12631-bib-0030] Williams, I.S. and Dixon, A.F.G. (2007) Life Cycles and Polymorphism In: Aphids as Crop Pests (edited by H. F. van Emden and R. Harrington). CABI International, Wallingford, U.K. pp. 69–87.

[imb12631-bib-0031] Wu, W. , Liang, X.L. , Zhao, H.Y. , Xu, T.T. and Liu, X.D. (2013) Special plant species determines diet breadth of phytophagous insects: a study on host plant expansion of the host‐specialized *Aphis gossypii* Glover. PLoS ONE, 8, e60832. 23580128 10.1371/journal.pone.0060832 PMC3620328

[imb12631-bib-0032] Yang, C. , Pan, H. , Liu, Y. and Zhou, X. (2015) Temperature and development impacts on housekeeping gene expression in cowpea aphid, *Aphis craccivora* (Hemiptera: Aphidiae). PLoS ONE, 10, e0130593. 26090683 10.1371/journal.pone.0130593 PMC4474611

[imb12631-bib-0033] Zanga, D. , Sanahuja, G. , Eizaguirre, M. , Albajes, R. , Christou, P. , Capell, T. *et al* (2018) Carotenoids moderate the effectiveness of a Bt gene against the European corn borer, *Ostrinia nubilalis* . PLoS ONE, 13, e0199317. 29990319 10.1371/journal.pone.0199317 PMC6038990

