# Supplementary material for: Transcriptional changes in the aphid species Myzus cerasi under different host and environmental conditions
Source: Insect Mol Biol. 2020 Jan 13;29(3):271–82. doi: 10.1111/imb.12631 (PMC7317760; doi:10.1111/imb.12631)
Supplement: Supplementary file 1 — Figure S1. Transcriptome differences between Myzus cerasi populations from different host environments. Genome‐wide analysis of M. cerasi transcriptional responses to interaction with primary host cherry (field) or secondary hosts cress and cleavers (both in controlled environment), and comparison to previously published tissue‐specific transcriptome of dissected heads and bodies (Thorpe, Cock, and Bos, 2016). (A) Clustering of transcriptional responses reveals that M. cerasi gene expression is different in populations from the different host environments and also that expression in head and body tissues can be separated based on these analyses. (B). Principal component analysis. The top three most informative principal components describe approximately 75% of the variation, and separate the both the host species interaction data as well as tissue‐specific data well. Figure S2. Validation of differential gene expression by Quantitative Rerverse Transcription PCR (RT‐qPCR). (A) Genes up‐regulated during cherry (field) vs. cleavers/cress (controlled environment) interactions in the Myzus cerasi population collected from location 1. (B) Genes up‐regulated during the cleavers/cress (controlled environment) vs. the cherry (field) interactions in the M. cerasi population collected from location 1. (C) Genes up‐regulated during cherry (field) vs. cleavers/cress (controlled environment) interactions in the M. cerasi population collected from location 2. (D) Genes up‐regulated during the cleavers/cress (controlled environment) vs. the cherry (field) interactions in the M. cerasi population collected from location 2. The validated genes up‐regulated during the cherry interactions were peroxidase (Mca14094‐Per), protein kinase (Mca07516‐PK), RNA binding (Mca07514‐RNAb), maltase (Mca25862‐Mal) and lactase (Mca19306‐Lac). Validated genes up‐regulated during the cress/cleavers interactions were venom protein (Mca05785‐Ven), uncharacterized protein (Mca06816‐UN), unknown protein ( [file IMB-29-271-s001.docx]

### Supplementary information

###
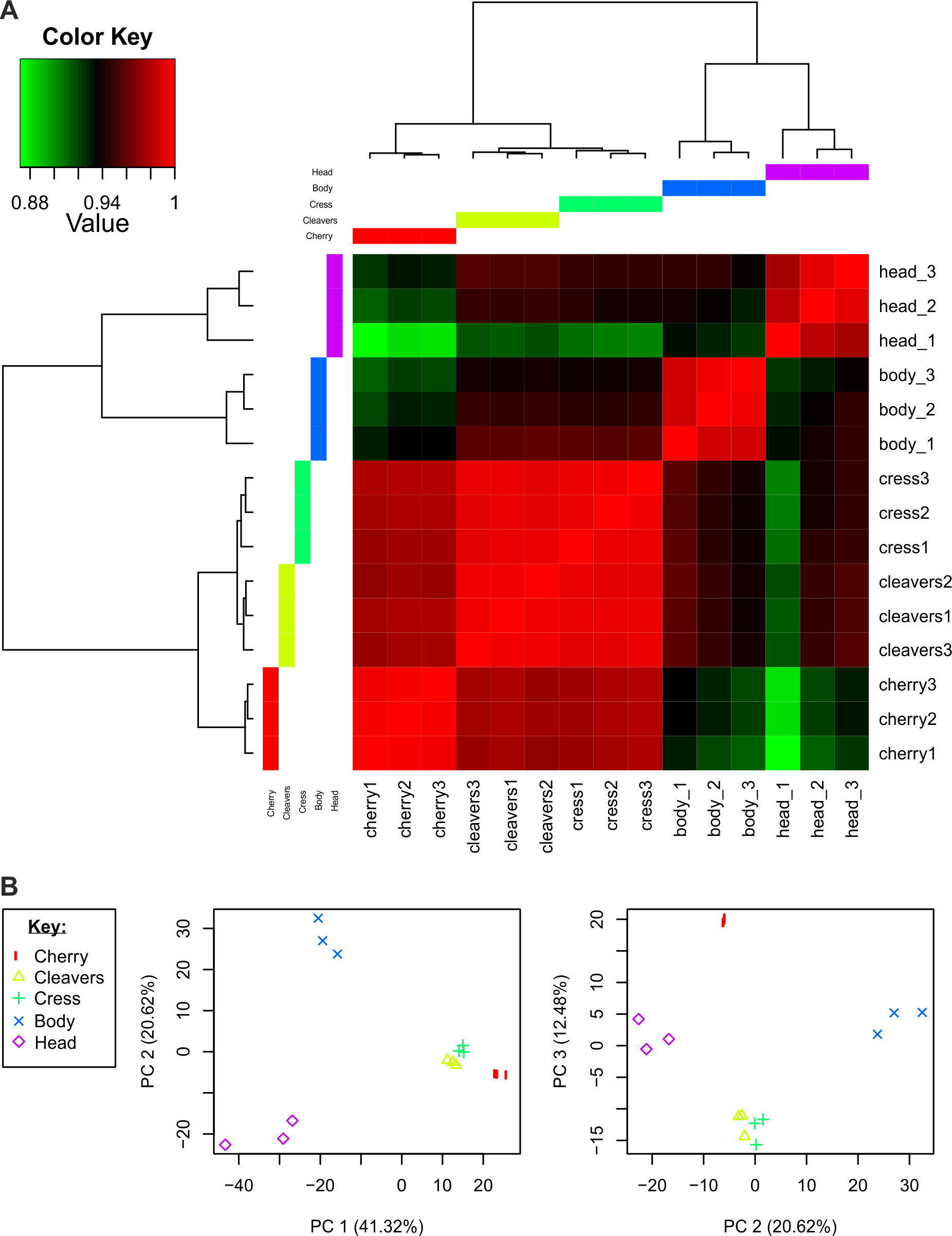


### Fig. S1. Transcriptome differences between *Myzus cerasi* populations from different host environments

Genome-wide analysis of *M. cerasi* transcriptional responses to interaction primary host cherry (field) or secondary hosts cress and cleavers (both in controlled environment), and comparison to previously published tissue-specific transcriptome of dissected heads and bodies (Thorpe, et al. 2016).

(A) Clustering of transcriptional responses reveals that *M. cerasi* gene expression is different in populations from the different host environments and also that expression in head and body tissues can be separated based on these analyses

(B). Principle component analysis. The top 3 most informative principle components describe approximately 75% of the variation, and separate the both the host species interaction data as well as tissue-specific data well.

**
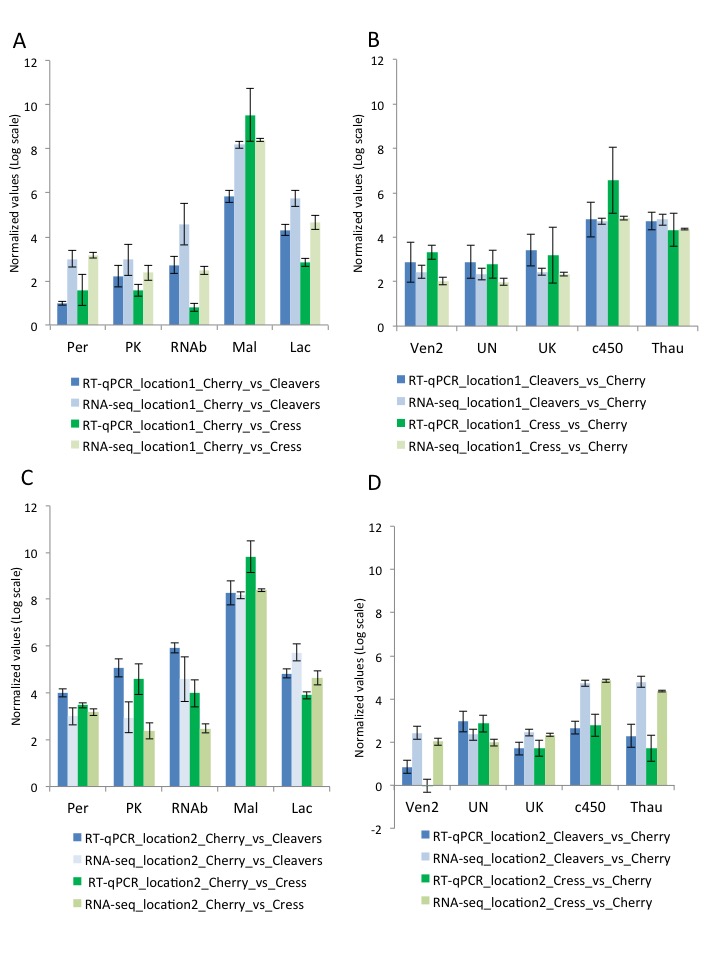
**

**Fig.S2.** Validation of differential gene expression by qRT-PCR

(A) Genes up-regulated during cherry (field) versus cleavers/cress (controlled environment) interactions in the *Myzus cerasi* population collected from location 1.

(B) Genes up-regulated during the cleavers/cress (controlled environment) versus the cherry (field) interactions in the *M. cerasi* population collected from location 1.

(C) Genes up-regulated during cherry (field) versus cleavers/cress (controlled environment) interactions in the *M. cerasi* population collected from location 2.

(D) Genes up-regulated during the cleavers/cress (controlled environment) versus the cherry (field) interactions in the *M. cerasi* population collected from location 2.

The validated genes up-regulated during the cherry interactions were peroxidase (Mca14094-Per), protein kinase (Mca07516-PK), RNA binding (Mca07514-RNAb), maltase (Mca25862-Mal) and lactase (Mca19306-Lac). Validated genes up-regulated during the cress/cleavers interactions were venom protein (Mca05785-Ven), uncharacterized protein (Mca06816-UN), unknown protein (Mca06864-UK), cytochrome 450 (Mca22662-c450) and thaumatin (Mca12232-Thau). Blue and green series represent RT-qPCR validation results and pale blue and pale green represent RNA-seq results.

Error bars indicate standard error.

**
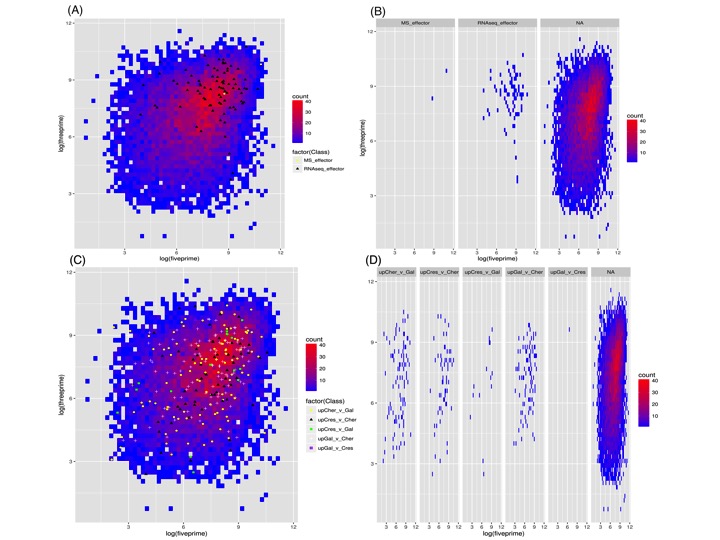
**

**Figure S3.** Heat maps graphically representing the LOG nucleotide distance from one gene to its neighboring genes in a 3’- and 5’ -direction. Various gene categories are colored and coded in the relevant keys. (A) and (B) Geneic distance heat map for predicted effectors, which were significantly further away from their neighboring genes and thus in gene sparse regions (Thorpe, et al. 2018). (C) and (D) Genic distances for differentially expressed genes identified in this study. These are not significantly further away from their neighboring genes in either 3’- or 5’-direction.
